# Acupuncture with manual and electrical stimulation for labour pain: a two month follow up of recollection of pain and birth experience

**DOI:** 10.1186/s12906-015-0708-2

**Published:** 2015-06-12

**Authors:** Linda Vixner, Lena B. Mårtensson, Erica Schytt

**Affiliations:** Department of Women’s and Children’s Health, Division of Reproductive Health, Karolinska Institutet, Retzius väg 13A, Karolinska Institutet, 171 77 Stockholm, Sweden; School of Health and Social Studies, Dalarna University, 791 88 Falun, Sweden; School of Health and Education, University of Skövde, P.O. Box 408, 541 28 Skövde, Sweden; Centre for Clinical Research Dalarna, Nissers väg 3, 791 82 Falun, Sweden

## Abstract

**Background:**

In a previous randomised controlled trial we showed that acupuncture with a combination of manual- and electrical stimulation (EA) did not affect the level of pain, as compared with acupuncture with manual stimulation (MA) and standard care (SC), but reduced the need for other forms of pain relief, including epidural analgesia. To dismiss an under-treatment of pain in the trial, we did a long-term follow up on the recollection of labour pain and the birth experience comparing acupuncture with manual stimulation, acupuncture with combined electrical and manual stimulation with standard care. Our hypothesis was that despite the lower frequency of use of other pain relief, women who had received EA would make similar retrospective assessments of labour pain and the birth experience 2 months after birth as women who received standard care (SC) or acupuncture with manual stimulation (MA).

**Methods:**

Secondary analyses of data collected for a randomised controlled trial conducted at two delivery wards in Sweden. A total of 303 nulliparous women with normal pregnancies were randomised to: 40 min of MA or EA, or SC without acupuncture. Questionnaires were administered the day after partus and 2 months later.

**Results:**

Two months postpartum, the mean recalled pain on the visual analogue scale (SC: 70.1, MA: 69.3 and EA: 68.7) did not differ between the groups (SC vs MA: adjusted mean difference 0.8, 95 % confidence interval [CI] −6.3 to 7.9 and SC vs EA: mean difference 1.3 CI 95 % −5.5 to 8.1). Positive birth experience (SC: 54.3 %, MA: 64.6 % and EA: 61.0 %) did not differ between the groups (SC vs MA: adjusted Odds Ratio [OR] 1.8, CI 95 % 0.9 to 3.7 and SC vs EA: OR 1.4 CI 95 % 0.7 to 2.6).

**Conclusions:**

Despite the lower use of other pain relief, women who received acupuncture with the combination of manual and electrical stimulation during labour made the same retrospective assessments of labour pain and birth experience 2 months postpartum as those who received acupuncture with manual stimulation or standard care.

**Trial registration:**

ClinicalTrials.gov: NCT01197950

## Background

Acupuncture involves the puncturing of the skin with thin sterile needles, at defined acupuncture points, that are then stimulated either manually or electrically. In manual acupuncture (MA) the needles are twisted back and forth by hand until a sensation of DeQi is achieved. In electro-acupuncture the needles are connected to a stimulator that delivers either high or low frequency impulses, or a combination of both [1]. In all Swedish labour units, acupuncture is available as an option to reduce women’s pain during labour, despite contradictory results from studies evaluating its effectiveness during labour. Acupuncture seems to help women manage labour pain and avoid pharmacological pain relief, though it is still unclear if acupuncture can reduce pain intensity [2, 3]. Some studies have found that acupuncture leads to a reduction of pain during labour [4, 5], whereas other studies have not [6–9]. Three studies, however, reported that acupuncture leads to a reduced use of pharmacological pain relief such as epidural analgesia and pethidin [6, 7, 10]. This lower frequency of use of pharmacological pain relief may reflect lower pain intensity due to the effects of acupuncture but it is also a possibility that it reflects insufficient treatment of pain.

This uncertainty raises questions about the long-term effect of acupuncture on the birth experience in general and on labour pain in particular. The birth experience is complex and affected by many factors such as expectations, support from the caregiver and the quality of the relationship between the caregiver and the woman, including the involvement in decision making [11]. Women’s experiences of a painful labour and birth are not only important during the process of labour and birth but they also have long-term consequences for women’s health and wellbeing. Women who remember their first birth as a negative experience at 2 months postpartum have fewer subsequent children and a longer interval before their next pregnancy [12]. A negative birth experience is also an important predictor of depressive symptoms during the first year of motherhood [13].

Some have found that the experience of childbirth and pain is highly correlated [14], also at 2 months after birth [15], while others have found that the care and support received during labour and birth is more important than pain for the birth experience [11]. It is claimed that labour pain is quickly forgotten, but most women who have been in labour describe the pain as the most intense they have ever experienced [16]. The interpretation of retrospective assessments of labour pain is difficult [16]. In the first few days after the birth, recollection of labour pain and satisfaction levels regarding pain relief received during labour may be influenced by a number of factors including analgesic drugs with an amnestic effect [16], high oxytocin levels affecting memory [17, 18], additional painful symptoms such as perineal pain and after pains and adverse birth outcomes [16], any of which may overshadow previous pain. In addition, assessments of pain during and after the birth may reflect different dimensions of pain. The in-labour assessments of pain seems to primarily reflect the sensory and affective dimensions, whereas recollection of pain mainly reflects the cognitive-evaluative aspects of pain [19]. Information about pain scores during labour and the recollection of pain are thus important but for different reasons; to optimise the support during labour *or* to optimise the postnatal support [14].

We have previously shown that acupuncture with manual stimulation or acupuncture with a combination of manual and electrical stimulation (in our study named EA) was not superior to standard care (SC) when pain was assessed prospectively on a Visual Analogue Scale (VAS) during labour (mean estimated pain was: SC 69.0; MA 66.4 and EA 68.5) [7]. However, women who received (EA) used other forms of pain relief, including epidural analgesia, to a lesser extent than those who received manual acupuncture alone (MA) or standard care (SC) [7]. There was no difference in satisfaction regarding pain relief between the groups the day after the birth (EA 81 %; MA 77 %; SC 74 %) [7].

The results from our previous study, however, raised some concern about the reduced use of epidural analgesia and other pain relief methods in the EA group. Even though the women’s needs for pain relief seemed to have been met to the same degree in the EA group as in the other two groups, we could not rule out the possibility that women in the EA group had received less pain relief than they actually needed. Blinded control interventions in acupuncture research are problematic as it is possible that these interventions have similar physiological effects to acupuncture itself in the activation of the endogenous opioid system [20, 21]. For this reason, this study was not blinded and this could have had an impact on the use of other pain relief methods. The decision to use epidural analgesia is not made independently by the woman in labour but rather in consultation with the care provider and in accordance with the local culture of the labour ward [22]. If the low frequency of use of epidural analgesia and other pain relief in the EA group was, in fact, due to influence from the midwives, this could have affected the woman’s experience of own involvement in the decision making and the midwife support, which is important for the birth experience [11]. This could also have affected the recollection of labour pain in a negative way [14]. Very little is known about the long-term effects of acupuncture on women’s recollection of labour pain and the birth experience. None of the studies on acupuncture mentioned above included a follow-up measurement of labour pain and only one included a follow-up of the birth experience at 2 months postpartum [6], where no differences in the birth experience were reported between the groups (acupuncture, transcutaneous electrical nerve stimulation [TENS] and standard care).

Given the lower frequency of use of pain relief among women receiving EA, we made a long-term follow up on the recollection of labour pain and the birth experience, and compared acupuncture with combined electrical and manual stimulation to 1) acupuncture with manual stimulation and 2) standard care. Our hypothesis was that despite the lower frequency of use of other pain relief, women who received EA would make similar retrospective assessments of labour pain and the birth experience 2 months after birth to women who received SC or MA.

## Methods

This study presents findings from secondary analyses of data collected for a randomised controlled trial conducted at two delivery wards in Sweden [7, 23]. The trial included 303 nulliparous women who were randomised into the following groups; manual acupuncture (MA), a combination of manual and electrical stimulation, i.e. electro-acupuncture (EA), or standard care without acupuncture (SC). The study protocol followed the CONSORT [24] and STRICTA [25] recommendations and the rationale of acupuncture was based on Western medical theories [26–28]. A full description of the study design [23] and the primary results have been published previously [7], and the trial was registered at ClinicalTrials.gov: NCT01197950.

Inclusion criteria for participation were: healthy nulliparous women with normal singleton pregnancies and a foetus in cephalic presentation admitted to the delivery ward in a latent or active phase of labour after a spontaneous onset of labour. Women were excluded if they had received any pharmacological pain relief within the 24 h prior to inclusion into the study with the exception of paracetamol, or if they were given oxytocin at the time point of allocation. Characteristics of the women at the time of giving birth are presented in Table 1.

**Table 1:** Characteristics of the women, use of pain relief, labour outcomes and infant data

	MA (*n* = 83)	EA (*n* = 87)	SC (*n* = 83)			
Characteristics of the women						
Age (years), mean (SD)	26.5 (4.8)	27.6 (4.6)	28.3 (5.0)			
Born in Sweden (%)	91.3	89.7	90.2			
Higher education (%)	35	44.8	54.2			
Single parent (%)	14.5	18.4	15.7			
Smoking 3 months prior to pregnancy (%)	23.0	19.5	19.7			
Body mass index in early pregnancy, mean (SD)	24.4 (5.0)	24.2 (3.8)	24.9 (4.1)			
Cervix dilatation at admission (cm), mean (SD)	3.6 (1.5)	4 (1.6)	3.6 (1.8)			
Membranes ruptured before admission (%)	30.5	28.7	33.3			
				MA vs. SC	EA vs. SC	MA vs. EA
Labour outcomes and pain relief						
				OR (CI)^a^	OR (CI)^a^	OR (CI)^b^
Nitrous Oxide (%)	95.1	95.4	93.8	1.89 (0.43–8.37)	1.52 (0.39–5.96)	0.80 (0.17–3.75)
Sterile water injections (%)	12.2	4.7	10.0	1.15 (0.42–3.14)	0.40 (0.11–1.40)	0.35 (0.10–1.17)
TENS (%)	14.5	12.6	48.1	0.17 (0.77–0.37)	0.16 (0.73–0.34)	0.94 (0.38–2.33)
Morphine (%)	10.8	1.2	6.3	1.87 (0.59–5.95)	0.17 (0.20–1.53)	0.09 (0.01–0.76)
Epidural analgesia (%)	61.4	46.0	69.9	0.62 (0.32–1.20)	0.35 (0.19–0.67)	0.57 (0.31–1.06)
Mode of Delivery						
Normal vaginal (%)	74.7	74.7	74.7	0.97 (0.46–2.02)	0.94 (0.46–1.91)	0.97 (0.48–1.99)
Instrumental vaginal (%)	16.9	19.5	12.0	1.52 (0.61–3.81)	1.93 (0.81–4.63)	1.27 (0.56–2.87)
Caesarean (%)	8.4	5.7	13.3	0.64 (0.23–1.79)	0.41 (0.14–1.26)	0.65 (0.20–2.14)
				HR (CI 95 %)^a^	HR (CI 95 %)^a^	HR (CI 95 %)^b^
Duration of labour (minutes) mean (SD) c	619 (378)	500 (319)	615 (398)	1.03 (0.75–1.41)	1.44 (1.06–1.97)	1.41 (1.03–1.91)
Infant data				OR (CI)^a^	OR (CI)^a^	OR (CI)^b^
Transferal to neonatal care unit (%)	3.6	11.5	4.9	0.91 (0.19–4.31)	2.82 (0.82–9.68)	3.11 (0.81–11.98)
				*p*	*p*	*p*
Apgar score <7 at 5 minutes (%)	1.2	2.3	0	1.00	0.68	0.69
Umbilical cord arterial pH, mean (SD)	7.3 (0.7)	7.2 (0.7)	7.3 (0.8)	1.00	0.52	0.45
Umbilical cord venous pH, mean (SD)	7.3 (0.7)	7.3 (0.8)	7.3 (0.6)	1.00	0.68	0.69
Head circumference (cm), mean (SD)	34.9 (1.4)	34.9 (1.3)	35 (1.3)			
Birth weight (grams), mean (SD)	3508 (410)	3590 (456)	3654 (493)			

*MA* Manual acupuncture, *EA* Electro-acupuncture, *SC* Standard care, *SD* Standard deviation, *OR* Odds Ratio, *HR* Hazard Ratio, *CI* 95 % Confidence interval, *SD* Standard Deviation, *TENS* Transcutaneous Electrical Nerve Stimulation

^a^SC is reference

^b^MA is reference, adjusted for age and education

^c^From first treatment to partus

The randomisation was computerised by the first author (LV) and conducted in blocks of 9, 12 and 15, which were varied randomly. After randomisation and when requesting pain relief, women in the MA and EA groups were treated with 13–21 needles at 3 bilateral distal points and 4–8 bilateral local points, all within the same somatic area as the cervix and uterus. A number of adequate acupuncture points were listed by the research team, and the choice of local and distal points was left to the midwife. The needles were inserted and stimulated manually until DeQi was achieved and thereafter stimulated at ten-minute intervals for 40 min. In the EA group, the needles were inserted and first stimulated manually until DeQi was achieved, then eight of the local needles were connected to an electrical stimulator which was set at a high frequency (80 Hz) stimulation and the women adjusted the intensity of the electrical stimulation themselves to a level just under the pain threshold. The decision regarding which local needles were to be connected to the stimulator was made by the midwife. The midwives’ training and experience of administering acupuncture during labour varied [23], and to assure that the intervention procedures were performed correctly we conducted a one-day study-specific course that included practical sessions in how to administer MA and EA. Women in the SC group received other forms of pain relief available on the delivery wards. After the first acupuncture treatment, women in the MA end EA groups had access to all types of pain relief available on the delivery wards including additional acupuncture treatments. Women in the SC group had access to all forms of pain relief with the exception of acupuncture. The use of obstetric pain relief methods that were administered during labour is presented in (Table 1). A different person (assistant nurse or midwife) from the one who administered the intervention assisted the women in the procedure of measuring pain and relaxation during labour. About two hours after the birth, the women were transferred to a postpartum ward, and were cared for by other midwives than in the labour ward. Two months postpartum the participants were requested to respond to a postal questionnaire, which included validated instruments or single item questions used in previous studies on the following:

*Recalled labour pain and relaxation 2 months after birth,* which was assessed by using a Visual Analogue Scale (VAS); a 100 mm horizontal ungraded line with two endpoints: ‘no pain’/‘relaxed’ (left) and ‘worst imaginable pain’/‘very tense’ (right). The VAS is a validated and commonly used instrument for assessing pain and has been used in previous studies of acupuncture for labour pain [4–6, 8, 10, 29] and pain recollection [30, 31].

*Pain difference* was defined as the difference between the highest pain assessment on VAS during labour (*peak pain*) and the assessment of pain at 2 months after the birth.

The experienced labour pain in relation to expectations, *pain worse than expected*, was measured by the question ‘Compared to your expectations, what was your experience of pain?’ and the response alternatives were dichotomised into ‘worse than expected’ (much worse than expected + worse than expected) and as expected/milder (as expected + milder than expected + much milder than expected) The overall assessment of *sufficient pain relief* was assessed by the question: ‘In summary, what is your assessment of all the pain relief you were given during labour?’ with the response alternatives: sufficient/insufficient.

The *experienced effect of acupuncture for reducing pain and increasing relaxation* was assessed by ‘In summary, what is your assessment of your acupuncture treatment for pain relief/relaxation?’ and the response alternatives were dichotomised into effective (very effective + rather effective) and ineffective (not very effective + not effective at all). In addition, a question asking whether the woman would *choose the same treatment in a forthcoming labour* or not (yes/no) was included.

*Specific emotions during labour*: The women were presented to a number of positive and negative emotions that may or may not have been experienced during labour and birth. They were asked to circle all the words that described emotions they had experienced during labour from the following list: Strong/ Weak/ Happy/ Sad/ Calm/ Frightened/ Alert/ Tired/ Secure/ Worried/ Involved/ Lonely/ Detached/ Independent/ Empowered/ Abandoned/ Determined/ Tense/ Trust in my own capacity/ Challenged/ Focused/ Panicked/ Disappointed/ Present. The words were coded as yes/no depending on the presence or absence of a circle [32]. Before commencement of this study, these words were tested on 64 women at the postnatal ward the day after giving birth, who were not included in the trial. We instructed them to circle the words describing their emotions during labour, and also to add emotions they had experienced that were not included on the list. This resulted in the addition of Disappointed and Present to the list.

*Summary of emotions during labour*: This was assessed with the question ‘In summary, how were your emotions during delivery’ with the response alternatives: ‘positive’ or ‘negative’.

*Overall birth experience* was assessed by a single item question which has been used in a number of previous studies; ‘How was your overall birth experience?’ and the response alternatives were dichotomised into positive (very positive + positive) and mixed/negative (mixed feelings + negative + very negative) [33–35].

*Depressive symptoms* were assessed by the Edinburgh Postnatal Depression Scale (EPDS) The EPDS was established to screen for postnatal depression and is a 10-item self-reported scale [36] and has been validated also in Sweden [37, 38]. Each item is scored on a scale from 0 to 3, giving a total minimum of 0 and maximum of 30, and scores ≥13 indicate depressive symptoms [38]. The scale rates depressive symptoms within the previous seven days.

*Perception of the midwife* was assessed by the question ‘In summary, what was your impression of your midwife?’ with the response alternatives: ‘positive’ or ‘negative’.

*Support from midwife during labour* was assessed by a single item question ‘Did your midwife give you the support you required during delivery?’ and the response alternatives were dichotomised into ‘Support to a high extent’ (yes, to a high extent) and ‘Not support to a high extent’ (yes, to a rather high extent + no, to a rather low extent + no, not at all).

### Statistics

The sample size calculation was based on the primary outcome which was women’s assessments of pain during labour, which has been described previously [7, 23].

Baseline characteristics are reported as means for continuous variables and percentages for discrete variables (Table 1). A generalised linear model (GLM) was performed to investigate possible associations between treatment (MA, EA, SC) and the following three outcomes: 1) recollection of pain/relaxation at 2 months after the birth, 2) the difference between peak pain and memory of pain at 2 months after the birth, and 3) the mean number of positive/negative emotions. In the model, adjustments were made for age and education, which statistically differed between the groups at the time of randomisation.

Associations between treatment and nine variables were analysed by means of logistic regression analyses and similar adjustments as in the GLM model were made. These variables were: 1) pain worse than expected, 2) sufficient pain relief, 3) would choose the treatment in a forthcoming labour, 4) acupuncture effective for reducing pain/relaxation, 5) positive birth experience, 6) overall positive emotions, 7) EPDS ≥13, 8) perception of midwife, and 9) support from midwife during labour. The results are reported as Odds Ratios (OR) with 95 % confidence intervals (CI).

### Ethics statement

Written informed consent was received from all participants included in the study. The study was approved by the Regional Ethical Review Board, Gothenburg, 15 May 2008, Dnr: 136–08.

## Results

**Fig. 1:**
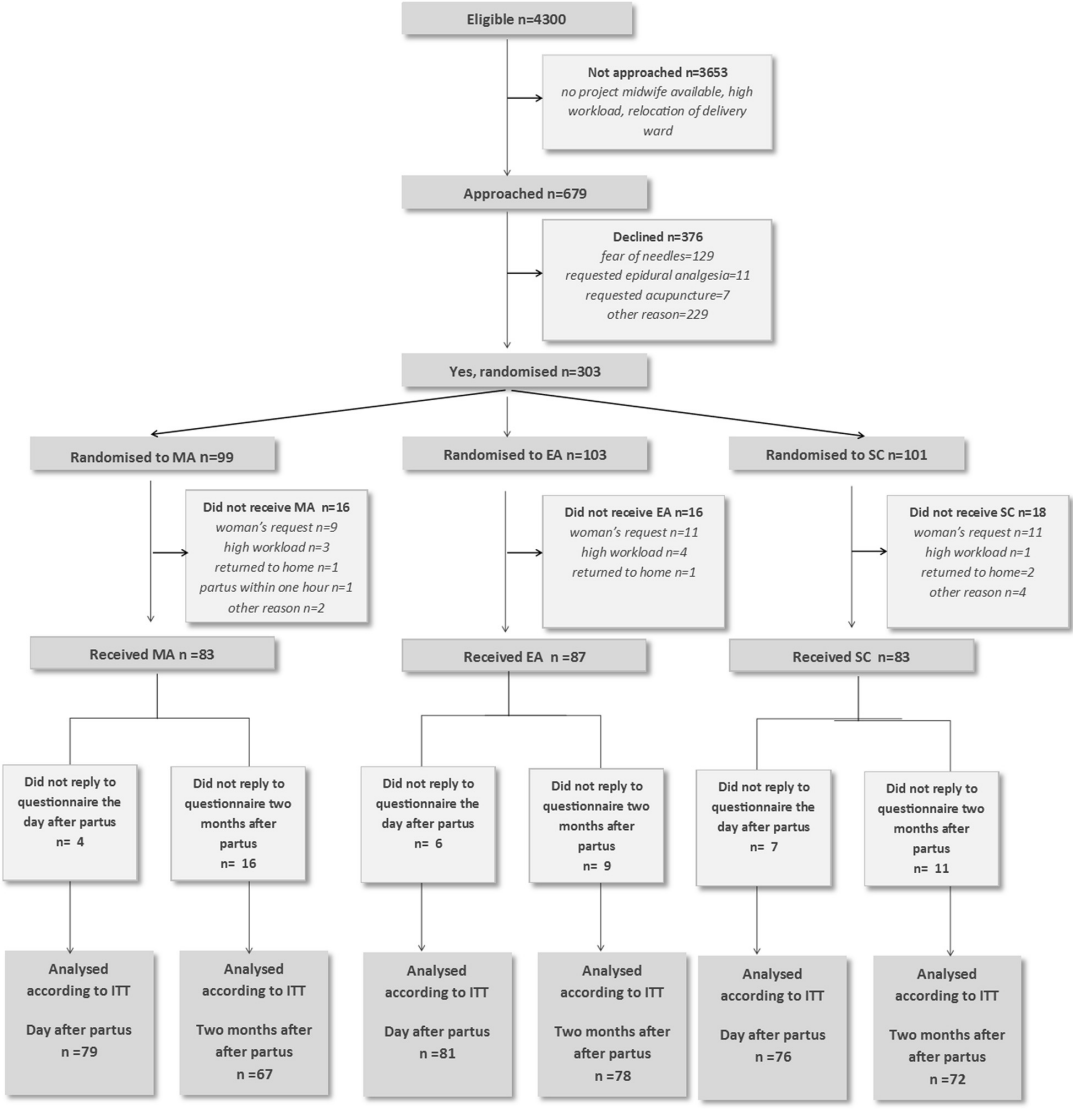
Flow chart of the study participants. MA = Manual acupuncture. EA = Electro-acupuncture. SC = Standard Care. ITT = Intention to treat

Recruitment and participation are presented in Fig. 1. Approximately 4300 women were eligible, 679 were informed and asked to participate in the study. A total of 303 consented to participate. The interventions were given to 253 women; MA 83, EA 87, and SC 83. The questionnaire 2 months postpartum was completed by 67 women in the MA group (81 %), 78 in the EA group (90 %), and 72 in the SC group (87 %). The mean number of days after birth for responding to the questionnaire was: MA 65.7 (SD 11.7), EA 68.3 (SD 17.5), and SC 69.2 (SD 14.5). There were no differences between the groups regarding Apgar score <7 at 5 min, transfer to neonatal intensive care unit or umbilical cord pH (Table 1).

The overall mean recalled labour pain on the VAS 2 months postpartum was similar in the groups, both the unadjusted mean scores and when adjusted for age and education (Table 2). The adjusted mean scores for recalled relaxation were also similar in the groups (MA 52.8; EA 53.1; SC 55.8). The mean differences were as follows: SC vs MA: mean difference 3.0 CI 95 % −5.3 to 11.2, SC vs EA: mean difference 2.7 CI 95 % CI −5.3 to 11.2, and MA vs EA: mean difference −0.3 CI 95 % −8.3 to 7.8.

**Table 2:** Experience of labour, acupuncture treatment and emotional wellbeing assessed at two months after birth

	MA	EA	SC	MA vs. SC^a^	EA vs. SC^a^	EA vs. MA^b^
	*n* = 67	*n* = 78	*n* = 72			
*Labour pain*				Mean difference (CI 95 %)^c^	Mean difference (CI 95 %)^c^	Mean difference (CI 95 %)^c^
Recalled labour pain, mean (SE)	69.3 (3.0)	68.7 (2.8)	70.1 (2.8)	0.8 (−6.3–7.9)	1.3 (−5.5–8.1)	0.5 (−6.4–7.4)
Peak pain (measured during labour), mean (SE)	81.6 (1.6)	83.2 (1.6)	85.8 (1.6)	4.1 (0.3–8.1)	2.6 (−1.2–6.4)	−1.6 (−5.3–2.2)
Difference between peak and recalled pain (SE)	11.7 (3.0)	14.1 (2.8)	13.7 (2.8)	2.0 (−5.1–9.2)	−0.4 (−7.2–6.4)	−2.4 (−9.3–4.5)
				OR (CI 95 %)^d^	OR (CI 95 %)^d^	OR (CI 95 %)^d^
Pain worse than expected (%)	42.4	42.7	47.1	0.8 (0.4–1.6)	0.8 (0.4–1.6)	1.0 (0.5–2.0)
Sufficient pain relief (%)	75.4	84.4	75.0	1.2 (0.5–2.9)	2.1 (0.9–4.9)	1.7 (0.7–4.0)
*Acupuncture treatment*						
Would choose the treatment in a forthcoming labour (%)	52.2	50.6				0.8 (0.4–1.5)
Effective for reducing pain (%)	34.3	50.7				1.8 (0.9–3.6)
Effective for relaxation (%)	47.7	51.4				1.1 (0.5–2.1)
*Psychological outcomes*				Mean difference (CI 95 %)^c^	Mean difference (CI 95 %)^c^	Mean difference (CI 95 %)^c^
No. positive emotions, mean (SE)	4.0 (0.4)	4.4 (0.4)	4.0 (0.4)	0.0 (−0.9–1.0)	−0.4 (−1.4–0.5)	−0.5 (−1.4–0.5)
No. negative emotions, mean (SE)	1.9 (0.2)	1.7 (0.2)	1.8 (0.2)	−0.1 (−0.7–0.5)	0.7 (−0.5–0.6)	0.2 (−0.4–0.7)
				OR (CI 95 %)^d^	OR (CI 95 %)^d^	OR (CI 95 %)^d^
Overall positive emotions (%)	87.9	84.6	81.9	1.6 (0.6–4.1)	1.3 (0.5–3.0)	0.8 (0.3–2.1)
Positive birth experience (%)	64.6	61.0	54.3	1.8 (0.9–3.7)	1.4 (0.7–2.6)	0.8 (0.4–1.5)
Depressive symptoms (EPDS ≥13) (%)	4.5	5.1	8.3	0.3 (0.1–1.7)	0.5 (0.1–2.1)	3.1 (0.6–16.2)

*SC* = Standard care, *MA* = Manual acupuncture, *EA* = Electro-acupuncture, *SE* = Standard Error, *OR* = Odds Ratio, *CI* = 95 % Confidence interval

^a^SC is reference

^b^MA is reference

^c^Analysed by a generalized linear model (GLM) and adjusted for age and education

^d^Analysed by logistic regression and adjusted for age and education

The change from the prospectively measured peak pain during labour to the recollection of labour pain at 2 months after birth (pain difference) was also similar in the groups. In all three groups, women assessed the pain intensity as lower 2 months after birth than they had during labour (Table 2).

The rates of the following were also the same in the groups: satisfaction with pain relief, worse pain than expected, overall birth experience, number of positive and negative emotions and depressive symptoms (Table 2).

Regardless of treatment, the vast majority of women had a positive overall experience of their midwife (MA 95.5 %; EA 97.4 %; SC 97.2 %), which was similar between the groups (SC vs MA: OR 0.5 (95 % CI 0.1 to 3.1), SC vs EA OR 1.0 (95 % CI 0.1 to 7.1), and MA vs EA: OR 2.0 (95 % CI 0.3 to 12.5). The experience of midwife support during labour and birth was also similar in the groups (MA 58.2 %; EA 73.1 %; SC 69.4 %): SC vs MA: OR 0.6 (95 % CI 0.3 to 1.3), SC vs EA OR 1.2 (95 % CI 0.6 to 2.5), and MA vs EA: OR 1.9 (95 % CI 1.0 to 3.9)).

## Discussion

Our hypothesis that despite their lower frequency of use of other methods of pain relief, women who received acupuncture with a combination of manual and electrical stimulation would make similar retrospective assessments of labour pain and birth experience as those who received acupuncture with manual stimulation or standard care was confirmed. The recalled labour pain (mean pain scores on the VAS), birth experience, satisfaction with pain relief, and also recalled emotions during labour were all similar between the groups.

Our concern that the lower frequency of use of epidural and other pain relief in the EA group was based on the possibility that midwives held preferences towards EA rather than on the women’s need for pain relief [22] could thus be reduced. In our previous publication we reported that labour pain did not differ between the groups when assessed prospectively *during* labour and the majority of women in all three groups were satisfied with their overall pain management the day after partus, regardless of treatment [7]. Retrospective assessments conducted only a few days post partus could have been influenced by analgesic drugs or other types of pain [16]. Assessments of different aspects of the birth are in general more nuanced and less positive when some time has passed, and women are more critical of the care provided for her. However, the present follow-up study confirms that the effect of the treatments did not differ in a longer perspective regarding the recollection of labour pain, the satisfaction with the overall pain relief, as well as the overall birth experience.

When interpreting these research results, it is important to bear in mind that pain assessments made during labour were made until an epidural analgesia was administered or up to the time point of partus. It has been suggested that the recollection of labour pain reflects labour pain at its peak [16, 39], which in this study occurred close to the last measurement. One could expect that women in the EA group would have reported higher pain scores than women in the SC and MA groups, both during labour and when asked 2 months later, as women who received EA used a lower frequency of epidural and consequently remained in the study longer and continued to make pain assessments in a later and more painful stage of labour than the other two groups. However, the effects of the various treatments did not differ, neither in the assessed peak pain nor in the recollection of the labour pain. Our findings suggest that the women in this group have managed labour pain more successfully. EA is a relatively time consuming intervention that requires a high level of attendance from the midwife in the labour room. Instructing women to adjust the intensity of the treatment also means spending extra time with them. However, the level of satisfaction with the midwife and her support was not higher in the EA group than in the other groups, and the overall assessment of emotions during labour (positive/negative) was similar between the groups. A more probable explanation is that the self-management nature of the treatment where the women adjusted the intensity of the electrical stimulation themselves, increased the women’s experience of control. Having an influence on decisions regarding one’s care and having a feeling of control are important factors in managing labour pain [11].

Another finding indicating that women in our study were *not* denied the pain relief they wished for was that women in the EA group did not have a higher rate of negative birth experiences than women in the MA and SC groups. Similar findings were reported in a Danish acupuncture study comparing the effect of acupuncture with TENS and standard care on long-term birth experiences [6]. Acupuncture reduced the frequency of use of pharmacological pain relief and there were no differences in birth experiences found between the groups at 2 months postpartum. A memory of severe labour pain at 2 months after birth is highly correlated with a negative childbirth experience [15]. Other important risk factors are experiencing a lack of control during labour and being dissatisfied with the level of involvement in making decisions about one’s care [11]. We found no other negative long-term effects of acupuncture, including serious effects such as depressive symptoms.

This is the first study on the long-term effects of acupuncture for labour pain and we used several outcome measures to ensure that women’s need for additional pain relief had been met. Most of the instruments and single item questions used in this study have been validated or used in previous studies of labour pain and birth experience. The sample size calculation was based on the primary outcome which was women’s assessments of pain during labour and not the secondary outcomes presented in this article, and we cannot dismiss the fact that we do not have enough power to detect differences on these outcomes. The response rate to the questionnaire 2 months after birth was fairly high in all three groups and there was a similar number of dropouts within the groups, indicating that the group allocation did not influence the dropout rate. The dropouts in the EA group did not differ from those who responded to the questionnaire, but drop-outs from the MA group had higher BMI, lower education and were not born in Sweden. Drop-outs in in the SC group were smokers to a higher extent. However, the cases were few and the drop-out would probably not affect the conclusions. In addition there were no differences in the use of EDA between the women who did complete the questionnaire or not. Altogether, the results in this paper correspond to the results from our previous study evaluating the primary outcome [7] and to the results from the Danish study [6].

## Conclusion

Despite the lower use of other pain relief, women who received acupuncture with the combination of manual and electrical stimulation during labour made the same retrospective assessments of labour pain and birth experience 2 months postpartum as those who received acupuncture with manual stimulation or standard care.
